# Editorial: Machine Learning Methodologies to Study Molecular Interactions

**DOI:** 10.3389/fmolb.2021.806474

**Published:** 2021-12-03

**Authors:** Artur Yakimovich, Arzucan Özgür, Tunca Doğan, Elif Ozkirimli

**Affiliations:** ^1^ Pharma International Informatics, Roche Products Limited, Welwyn Garden City, United Kingdom; ^2^ Department of Computer Engineering, Bogazici University, Istanbul, Turkey; ^3^ Department of Computer Engineering, Hacettepe University, Ankara, Turkey; ^4^ Pharma International Informatics, F. Hoffmann-La Roche AG, Basel, Switzerland

**Keywords:** machine learning, molecular interactions, protein, biomolecule, DNA, interaction prediction

The cell is a busy place with proteins, DNA, RNA, metabolites and other molecules interacting with each other with orchestral precision. Disease states arise when this precision is lost for intracellular interactions or when external entities, such as virus particles, interact with intracellular molecules and disrupt this precision. As such, the study of molecular interactions is a huge area of focus for experimental and computational biologists alike.

Recognising the ever increasing uptake of Machine Learning (ML) in biomedical research, in this research topic, our focus was on the use of computational methodologies and ML approaches to examine molecular interactions. While experimental approaches such as structure determination of multimolecular complexes using X-ray crystallography or cryoEM are often the gold standard in studying intermolecular interactions, computational approaches are advantageous both because they are faster and less costly than experimental approaches and because some molecular interactions are neither easy nor feasible to study experimentally. In this special issue, the questions that the authors aimed to address ranged from understanding interactions at the residue or atomic level Karakulak et al.; Wang et al. to the cellular level Kyrilis et al.

The authors used a multitude of data sources and their combinations highlighting the value of multimodal analysis. Both sequence and structure-based predictors of specificity-determining residues in protein complexes were evaluated in the study of Karakulak et al. The authors proposed that the use of either approach by itself is not sufficient to accurately identify these residues, and new methods combining the advantages of both sequence and structure centric approaches are required. Protein interaction sites were identified by combining protein sequence and structure based information Wang et al., a reduced representation of proteins was built by molecular dynamics simulation data Errica et al., and genomic data was used to build an alternative splicing gene signature for cancer prognosis Zhao et al. Additionally, Kutlay and Aydin Son combined microRNA, mRNA, and DNA methylation data to build a metastasis model for melanoma cancer.

The articles in this issue have used ML methodologies ranging from shallow Support Vector machines (SVM) to deep learning based Graph Neural Networks (GNN) with success in interaction prediction, molecular representations and disease modeling. Two articles used graph neural networks powered by GPU. Wang et al. proposed a GNN based docking decoy evaluation score to identify near-native complex structures. By using an attention and gate-augmented mechanism, they captured the interaction pattern at the interface. A deep graph network enhanced sampling approach was proposed by Errica et al. to identify the coarse grained representation of proteins with minimal information loss. The mapping entropy provided information about the information loss due to mapping to a lower dimensional space. The authors used deep learning to accelerate mapping entropy calculation followed by Wang-Landau sampling to explore the mapping space of a molecule. This physics based coarse grained description of the molecular structure allowed the calculation of various properties by considering the dynamic nature of biomolecules.

Predicting interactions or interaction sites is not sufficient to predict the presence or absence of a potential disease state. Zhao et al. used alternative splicing signature as a predictor of non-small cell lung cancer prognosis using multivariate Cox regression. Going beyond the prediction of host-pathogen interactions, Karabulut et al. constructed an ML-based infection prediction model that predicts whether adenoviral infection can happen in a host, using multiple types of input features including host-pathogen PPIs and taxonomic preferences. Kutlay and Aydin Son built a prediction model for metastasis in melanoma. Arici and Tuncbag assessed the performance of various network reconstruction approaches over the use cases of reconstructing the Notch signaling and the glioblastoma (GBM) disease pathways, using different reference human interactome datasets, and showed that the performance is highly dependent on the source data. This study showed that the quality and coverage of the input data can be at least as important as the utilised algorithm when studying molecular interactions.

Perhaps one of the best ways to illustrate the versatility of ML methodologies when applied to molecular interactions is to demonstrate that such application may be performed both in a bottom-up and a top-down fashion. Two examples of such demonstrations in our research topic are the review article by Zrimec et al. and a perspective article by Kyrilis et al. The former explored the representation learning application to the central molecular dogma, i.e. learning biological molecules and their interactions from the genetic code (DNA to RNA to protein sequences). The latter reviewed studies utilizing machine learning approaches to analyze native cell extract as a source of experimental data on higher order molecular interactions. While the problem at hand of Zrimec and colleagues seems much more well studied, they presented a convincing case of innovative approaches in the field. For example, the authors reviewed a body of literature demonstrating that deep neural networks can automatically learn regulatory grammar through utilization of convolutional or recurrent neural networks. While these methods are widely applied in fields like Computer Vision and Natural Language Processing, their application to the genetic code gained popularity only recently. At the same time, Kyrilis and co-authors discussed the top-down approaches, which look at relatively noisy data sources to provide rich information about the inner workings of the cell through techniques such as cryo-electron microscopy and structural proteomics. In their perspective article they made a convincing case for ML methods being necessary and sufficient to tie together these modalities. Authors noted that inspired by Computer Vision, the tool-of-choice for cryo-electron microscopy is convolutional neural networks. Hence, it is this family of algorithms that receives the most attention from the researchers working on cell extracts to devise higher order molecular interactions.

Altogether, these studies serve as a great demonstration of the level of ML penetration into the study of Molecular Interactions. With the significantly elevated performance of deep learning-based single-chain protein structure predictors such as AlphaFold (Jumper et al., 2021) and RosettaFold (Baek et al., 2021), the focus has now been shifting to the accurate prediction of protein complex structures (Evans et al., 2021). The advances in cryoEM imaging, single cell imaging, proteomics (Piazza et al., 2020) methodologies also open new avenues for analyzing interactions in their native environments. It is clear that ML approaches to study molecular interactions are rapidly gaining traction but we, the Editors of this Research Topic, believe that the most exciting applications of ML to this domain are yet to be published. We look forward to reading the future research in this field.
